# Influence of early temperature trajectories on clinical outcomes in traumatic brain injury: a multicenter validation study using machine learning

**DOI:** 10.1186/s40001-025-03587-z

**Published:** 2025-12-02

**Authors:** Yunuo Zhao, Tao Zhang, Xi Zhong, Xuelei Ma

**Affiliations:** 1 Department of Biotherapy, Cancer Center and State Key Laboratory of Biotherapy, West China Hospital, Sichuan University, Chengdu, Sichuan China; 2 Department of Intensive Care Unit, West China Hospital, Sichuan University, Chengdu, Sichuan China

**Keywords:** Traumatic brain injury, Body temperature, Latent class mixed model (LCMM), Machine learning

## Abstract

**Objectives:**

Temperature management is a critical intervention to mitigate secondary injury in patients with traumatic brain injury (TBI). This study, based on the MIMIC database and externally validated using the eICU database, analyzed early 24-h temperature trajectories of TBI patients after ICU admission to investigate their association with clinical outcomes.

**Methods:**

Latent Class Mixed Model (LCMM) was employed to classify the 24-h temperature trajectories of TBI patients following ICU admission. Logistic regression models were constructed based on univariate selection, Boruta feature selection, and all variables to evaluate mortality risk across trajectory subtypes. Subgroup analyses were also performed. Furthermore, machine learning models were constructed using variables jointly selected by LASSO and Boruta, with multiple algorithms (Random Forest, XGBoost, LightGBM, logistic regression, SVM, and KNN) compared against traditional severity scores via DeLong’s test.

**Results:**

A total of 3249 TBI patients from the MIMIC database and 3246 patients from the eICU database were included, with temperature trajectories categorized into three distinct classes. According to the full-variable logistic model, patients in Class 1 and Class 3 exhibited significantly worse prognosis compared to Class 2 (*p* < 0.001). Sensitivity analyses yielded consistent results. Notably, the Random Forest model demonstrated superior predictive performance compared with conventional severity scores, such as SAPS II and OASIS.

**Conclusions:**

Temperature trajectories within the first 24 h of ICU admission are associated with clinical outcomes in TBI patients. Early identification of temperature trajectory subtypes facilitates timely recognition of high-risk patients with poor prognosis, enabling personalized temperature management strategies.

**Supplementary Information:**

The online version contains supplementary material available at 10.1186/s40001-025-03587-z.

## Background

Traumatic brain injury (TBI) is one of the leading causes of death and disability worldwide, particularly among critically ill patients in intensive care units (ICUs). An estimated 50 million TBI cases occur globally each year, imposing a substantial public health burden [1]. Patient outcomes following TBI are largely influenced by both the primary injury sustained at the moment of impact and the subsequent cascade of secondary injuries, including inflammatory responses, cerebral edema, metabolic dysfunction, and oxidative stress [2]. Neuroprotective strategies targeting secondary injury have become a central focus in the management of TBI [3].

In the ICU setting, temperature management is a key neurocritical care intervention aimed at mitigating secondary brain injury by controlling thermal homeostasis. Evidence suggests that secondary brain damage can occur within the first 24-h post-injury, underscoring the clinical importance of monitoring early temperature fluctuations [4]. Although the significance of temperature regulation is well-recognized, previous studies have primarily relied on temperature measurements at single timepoints, failing to capture the dynamic nature of early temperature trajectories and their potential prognostic implications. To address this, several statistical approaches have been developed for trajectory analysis using longitudinal data. Among them, latent class mixed modeling (LCMM) enables classification of patients into unobserved subgroups based on time-varying data [5]. This method has been applied to survival studies across diverse populations, including patients with esophageal cancer [6], sepsis [7, 8], and those undergoing peritoneal dialysis [9]. However, few studies have investigated temperature trajectories in TBI patients, and their prognostic implications remain unclear.

This study aims to employ LCMM approach to identify distinct 24-h temperature trajectories in ICU-admitted TBI patients using the MIMIC database, and to explore their associations with clinical outcomes. External validation will be conducted using the eICU database to enhance the robustness and generalizability of the findings. On this basis, we further used ICU mortality as the primary outcome and applied multiple machine learning methods to construct and compare predictive models. This research may offer new insights and evidence-based support for personalized temperature management and early prognostic assessment in TBI care.

## Methods

### Data source

Data for this study were obtained from the MIMIC-IV (version 3.1) database, with external validation using the eICU Collaborative Research Database (version 2.0). One of the authors completed the required training and was granted access to the databases (Record ID: 61575775). To protect patient privacy, all identifiers were replaced with randomly generated codes. As the data are de-identified, the requirement for ethical approval and informed consent was waived by West China Hospital, Sichuan University.

In the MIMIC-IV database, temperature measurements were obtained from multiple sites, including oral (80.8%), axillary (6.0%), esophageal (4.0%), rectal (3.5%), temporal (1.5%), blood (0.7%), and tympanic (0.1%), with 3.4% of measurements lacking site information. Oral temperature constituted the majority of records. In the eICU database, temperature sites included oral (20.9%), axillary (10.6%), core (10.1%), rectal (3.8%), tympanic (2.8%), bladder (2.7%), and temporal (1.4%), with 42.9% lacking site information.

### Study population

**Fig. 1:**
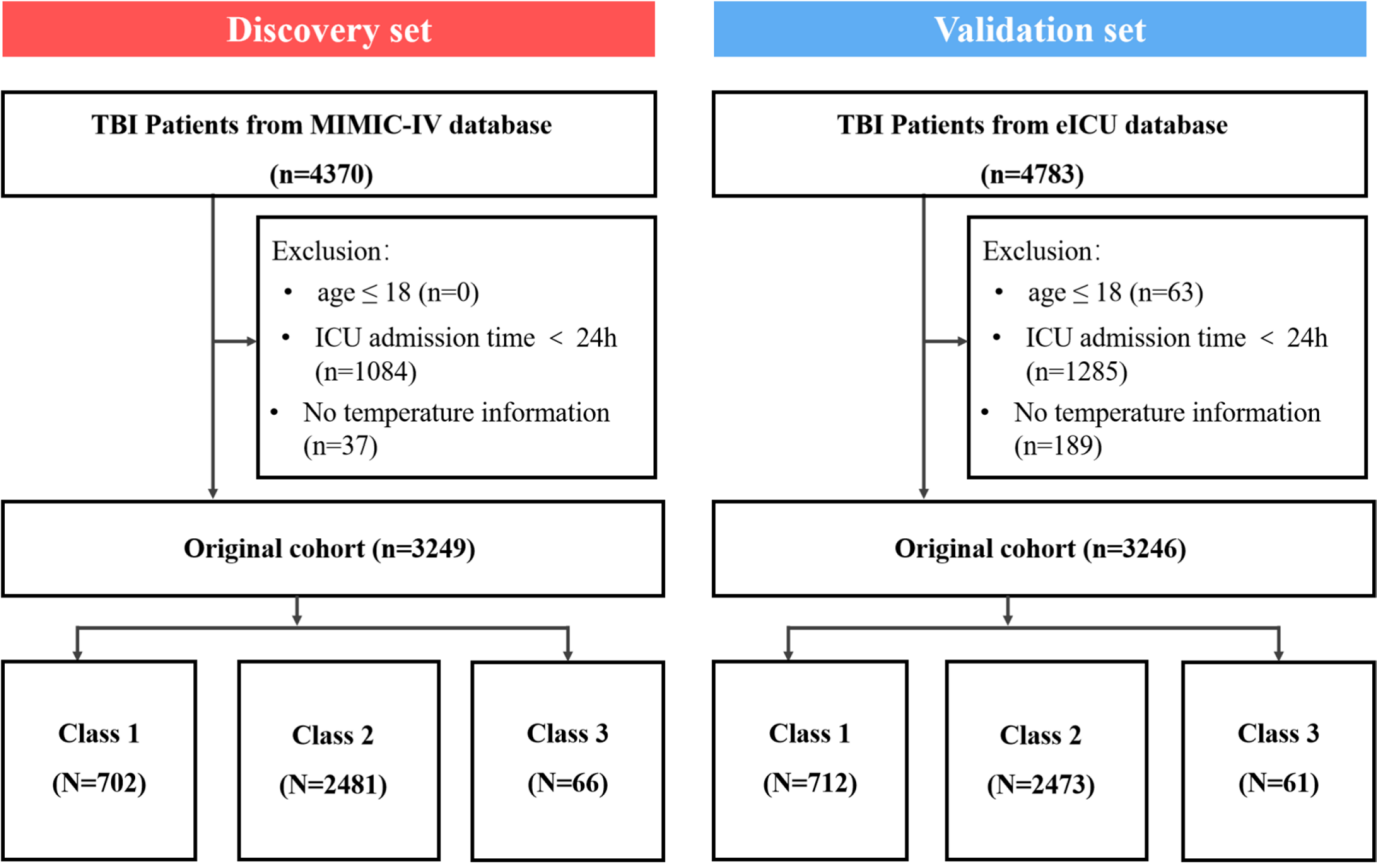
Specific process of data extraction

TBI patients were identified using ICD-9 and ICD-10 codes (Table S1). We excluded individuals younger than 18 years, those with fewer than three temperature measurements within the first 24 h of ICU admission, and those with an ICU stay of less than 24 h. A total of 3249 patients from the MIMIC database and 3246 patients from the eICU database were included. The detailed inclusion and exclusion process is shown in Fig. 1. Clinical data were extracted using PostgreSQL and SQL queries. Tables S2 and S3 summarize the baseline characteristics of the included patients. The primary outcomes were in-hospital mortality and ICU mortality; 28-day mortality was considered a secondary outcome.

### Data presentation

Categorical variables were expressed as frequencies and percentages, and group comparisons were performed using Pearson’s chi-square test or Fisher’s exact test, as appropriate. Continuous variables were summarized as mean ± standard deviation (SD) or median with interquartile range (IQR), depending on their distribution, and compared using Student’s *t* test or the Mann–Whitney *U* test. Missing values were addressed through multiple imputation, and variables with more than 40% missing data were excluded from the analysis.

### Latent class mixed model (LCMM)

We applied the LCMM using the lcmm package in R [10] to classify 24-h temperature trajectories among TBI patients in the MIMIC database. Models with 2 to 5 classes were evaluated, and the optimal number of classes was determined based on log-likelihood, entropy, Akaike Information Criterion (AIC), Bayesian Information Criterion (BIC), sample-adjusted BIC (SABIC), and the proportion of patients in each class. A minimum proportion threshold of 2% was predefined to ensure the robustness and clinical interpretability of the identified trajectories. For external validation using the eICU database, the pre-established LCMM parameters from the MIMIC model were applied. The predictClass() function was used to assign each patient in the eICU cohort to the most probable trajectory class based on posterior probabilities. Kaplan–Meier analysis was further performed to explore the association between trajectory classes and clinical outcomes.

### Variable selection

Candidate variables were initially screened using the Boruta algorithm [11], which evaluates variable importance based on Z-scores and classifies them as rejected (red), tentative (yellow), or confirmed (green). Variables classified as rejected or tentative were excluded from subsequent analyses, retaining only confirmed variables for sensitivity analyses. To further simplify the model and enhance predictive performance, Least Absolute Shrinkage and Selection Operator (LASSO) regression was also applied. After excluding clinical indicators such as the SOFA and GCS scores to avoid model redundancy, variables identified by both Boruta and LASSO were incorporated into the construction and analysis of the machine learning models.

### Sensitivity and subgroup analyses

To evaluate the robustness of our findings, sensitivity analyses were conducted using data from both the MIMIC and eICU databases. Specifically, we performed univariate logistic regression analyses, followed by multivariate logistic regression models adjusted either for all covariates or for covariates selected by the Boruta algorithm. Variables identified through these approaches were incorporated into the respective models to assess the stability and reproducibility of the associations between 24-h temperature trajectories and clinical outcomes across different model specifications and data sets. In addition, subgroup analyses were performed to examine the relationship between 24-h temperature trajectories and clinical outcomes across different patient subgroups.

### Machine learning

Using variables jointly selected by Boruta and LASSO, the MIMIC database served as the training set, with the eICU database used for external validation. Multiple machine learning algorithms—including Random Forest, XGBoost, LightGBM, Logistic Regression, Support Vector Machine (SVM), and K-Nearest Neighbors (KNN)—were employed to construct predictive models for ICU mortality risk. Feature contributions to model predictions were visualized using SHAP values. Model performance was assessed via receiver operating characteristic (ROC) curves and the corresponding area under the curve (AUC), with DeLong’s test applied to compare predictive performance across models.

## Results

### Trajectory grouping based on the LCMM model

After fitting and comparing LCMM models with varying numbers of latent classes, we observed a progressive decline in AIC, BIC, and SABIC values as the number of classes increased from 1 to 5, indicating improved model fit. However, the five-class and four-class model included a subgroup with a very small proportion, which undermined the model’s stability and interpretability.

In contrast, the three-class model provided an optimal balance, with relatively low information criteria (AIC = 47,540.04; BIC = 47,613.07; SABIC = 47,574.94) and a high entropy value (0.90) while maintaining reasonable class proportions. Therefore, considering model fit, classification quality, and interpretability, the three-class solution was ultimately selected as the best-fit model (Table S4). Moreover, the average posterior probabilities for all three classes exceeded 80%, supporting the reliability of the classification (Table S5).

**Fig. 2:**
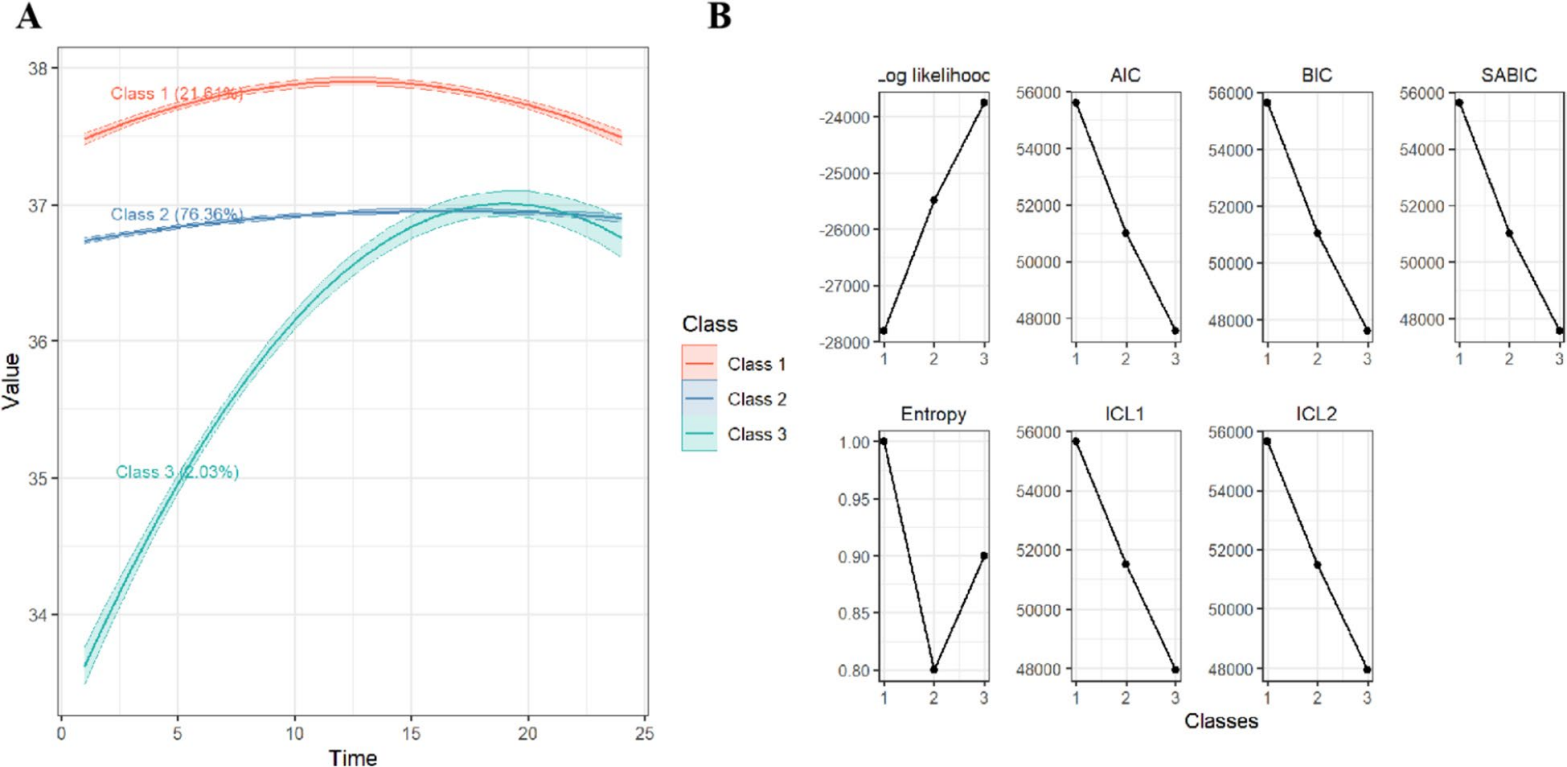
Latent class mixed model (LCMM)–based classification of 24-h temperature trajectories and model fit statistics in TBI patients. **A** Classification of 24-h temperature trajectories in TBI patients. **B** Model evaluation metrics, including AIC, BIC, CAIC, SABIC, HQIC, entropy, ICL1, and ICL2, used to determine the optimal number of classes

The 24-h temperature trajectories of TBI patients in the MIMIC database and their class proportions are shown in Fig. 2:

Class 1 (red, stable, high, 21.61%): Initially elevated, then slightly declines, overall remaining at a high level.

Class 2 (blue, stable, normal, 76.36%): Slight increase, remains within the normal range, with minimal fluctuations.

Class 3 (green, increasing, low, 2.03%): Marked rise from a low baseline to a peak, followed by a slight decline, with the greatest variability.

### Baseline characteristics

In both databases, patients in Class 2 exhibited the lowest SOFA scores and the highest Charlson comorbidity scores (*p* < 0.001). Regarding therapeutic interventions, this group had the lowest proportions of mechanical ventilation, propofol administration, and vasopressor use (*p* < 0.001). In addition, the incidence of sepsis was lowest in Class 2 (*p* < 0.001). Laboratory findings indicated that Class 2 patients had the lowest white blood cell counts, whereas Class 3 patients displayed the highest partial pressure of carbon dioxide (PaCO₂) and the lowest pH values (*p* < 0.01). Detailed information is provided in Tables S2 and S3.

### Clinical outcomes and sensitivity analyses

**Fig. 3:**
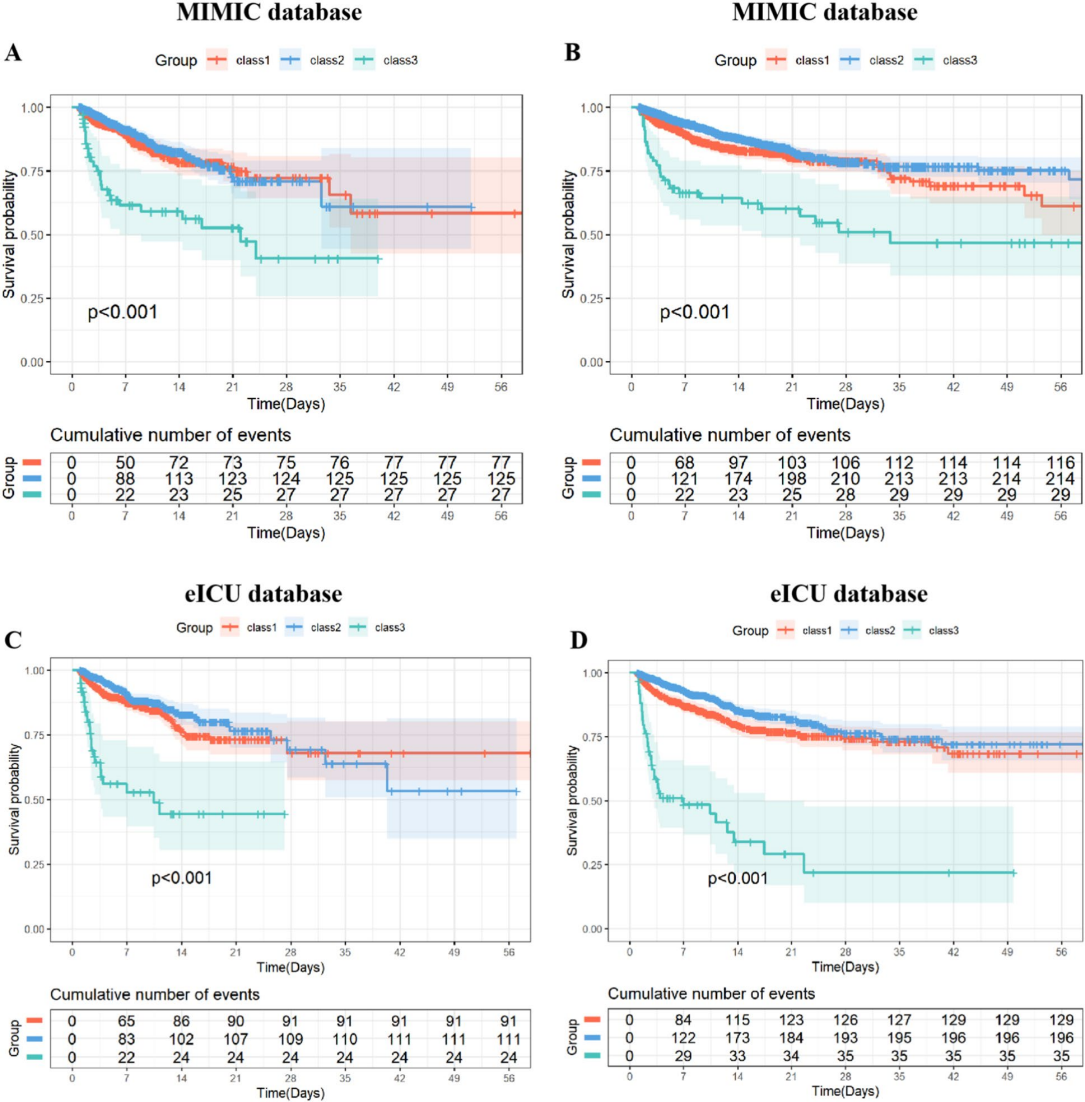
Kaplan–Meier analysis of clinical outcomes between groups in the MIMIC and eICU database. Kaplan–Meier analysis of ICU mortality between groups in the MIMIC (**A**) and eICU (**C**) database. Kaplan–Meier analysis of in-hospital mortality between groups in the MIMIC (**B**) and eICU (**D**) database

Kaplan–Meier analysis revealed that in the MIMIC database, Class 3 was associated with the highest 28-day, ICU, and in-hospital mortality rates (*p* < 0.001) (Fig. 3; Figure S1), and similar patterns were observed in the eICU database (*p* < 0.001) (Fig. 3).

**Fig. 4:**
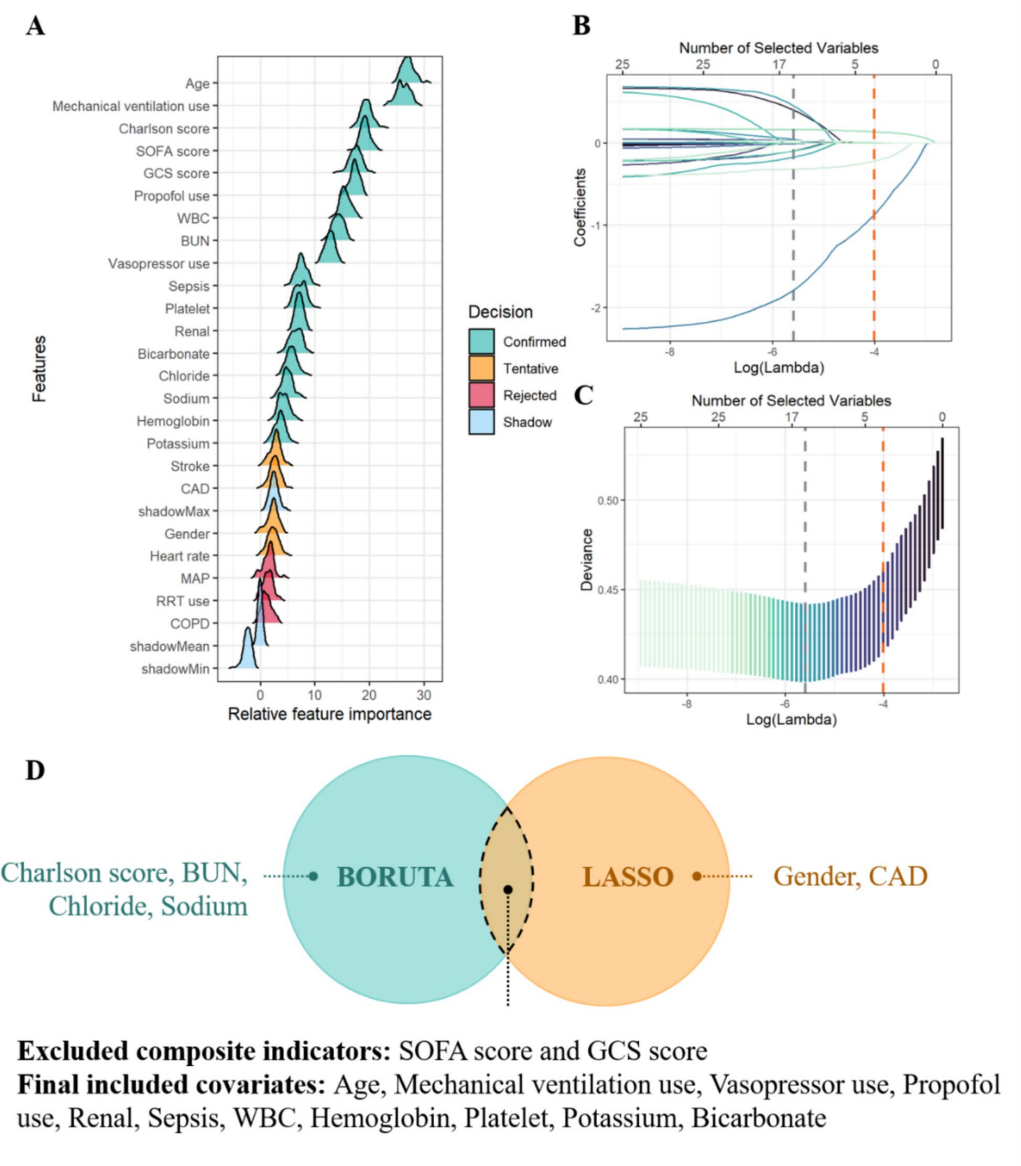
Variable selection. **A** Feature selection in MIMIC database using the Boruta algorithm. **B** Overview of LASSO coefficients for 25 candidate variables. **C** Parameter tuning of the LASSO model via tenfold cross-validation to determine the optimal lambda. **D** Intersection of Boruta- and LASSO-selected variables subsequently included in the machine learning models

Consistent results were obtained across both data sets using univariate screening, Boruta feature selection (Fig. 4A) (Figure S2), and multivariable logistic regression models incorporating all covariates. These analyses consistently demonstrated that patients in Class 2 had the most favorable outcomes, followed by those in Class 1, while Class 3 was associated with the poorest prognosis (Table 1).

**Table 1:** Primary and secondary outcome analyses with different models

	Class 2	Class 1			Class 3		
		OR	95% CI	*p* value	OR	95% CI	*p* value
MIMIC database							
28-day mortality							
Univariate Logistic Regression	Ref	1.49	1.19, 1.86	< 0.001	5.38	3.25, 8.84	< 0.001
Multivariate logistic model adjusted with all covariates	Ref	1.49	1.13, 1.96	< 0.01	4.91	2.60, 9.24	< 0.001
Multivariate logistic model adjusted with covariates selected by Boruta algorithm	Ref	1.56	1.19, 2.05	< 0.01	4.87	2.58, 9.16	< 0.001
ICU mortality							
Univariate Logistic Regression	Ref	2.32	1.72, 3.12	< 0.001	13.0	7.68, 21.9	< 0.001
Multivariate logistic model adjusted with all covariates	Ref	1.82	1.27, 2.58	< 0.01	6.46	3.35, 12.3	< 0.001
Multivariate logistic model adjusted with covariates selected by Boruta algorithm	Ref	1.79	1.26, 2.53	< 0.01	6.52	3.40, 12.4	< 0.001
In-hospital mortality							
Univariate Logistic Regression	Ref	2.09	1.63, 2.66	< 0.001	8.26	4.95, 13.7	< 0.001
Multivariate logistic model adjusted with all covariates	Ref	1.90	1.41, 2.57	< 0.001	5.96	3.14, 11.3	< 0.001
Multivariate logistic model adjusted with covariates selected by Boruta algorithm	Ref	1.96	1.46, 2.63	< 0.001	5.82	3.07, 11.0	< 0.001
eICU database							
ICU mortality							
Univariate Logistic Regression	Ref	3.12	2.33, 4.17	< 0.001	13.8	7.90, 23.7	< 0.001
Multivariate logistic model adjusted with all covariates	Ref	1.54	1.08, 2.19	< 0.05	2.39	1.20, 4.70	< 0.05
Multivariate logistic model adjusted with covariates selected by Boruta algorithm	Ref	1.51	1.07, 2.13	< 0.05	2.42	1.23, 4.71	< 0.05
In-hospital mortality							
Univariate Logistic Regression	Ref	2.56	2.01, 3.26	< 0.001	16.1	9.48, 27.7	< 0.001
Multivariate logistic model adjusted with all covariates	Ref	1.41	1.04, 1.91	< 0.05	3.71	2.92, 10.5	< 0.001
Multivariate logistic model adjusted with covariates selected by Boruta algorithm	Ref	1.40	1.04, 1.88	< 0.05	4.07	2.14, 7.83	< 0.001

### Subgroup analyses

The results of the subgroup analyses are presented in Figure S2. For ICU and in-hospital mortality, Class 3 demonstrated worse outcomes compared with Class 2 across most subgroups, except for patients with a SOFA score < 5 or a GCS score ≤ 8. The difference in ICU mortality between Class 2 and Class 1 was observed only in patients who did not receive propofol, were free of sepsis, or were aged ≥ 65 years. In the analysis of 28-day mortality, Class 3 exhibited higher mortality than Class 2 in all subgroups except among patients receiving albumin (Figure S3).

### Machine learning

**Fig. 5:**
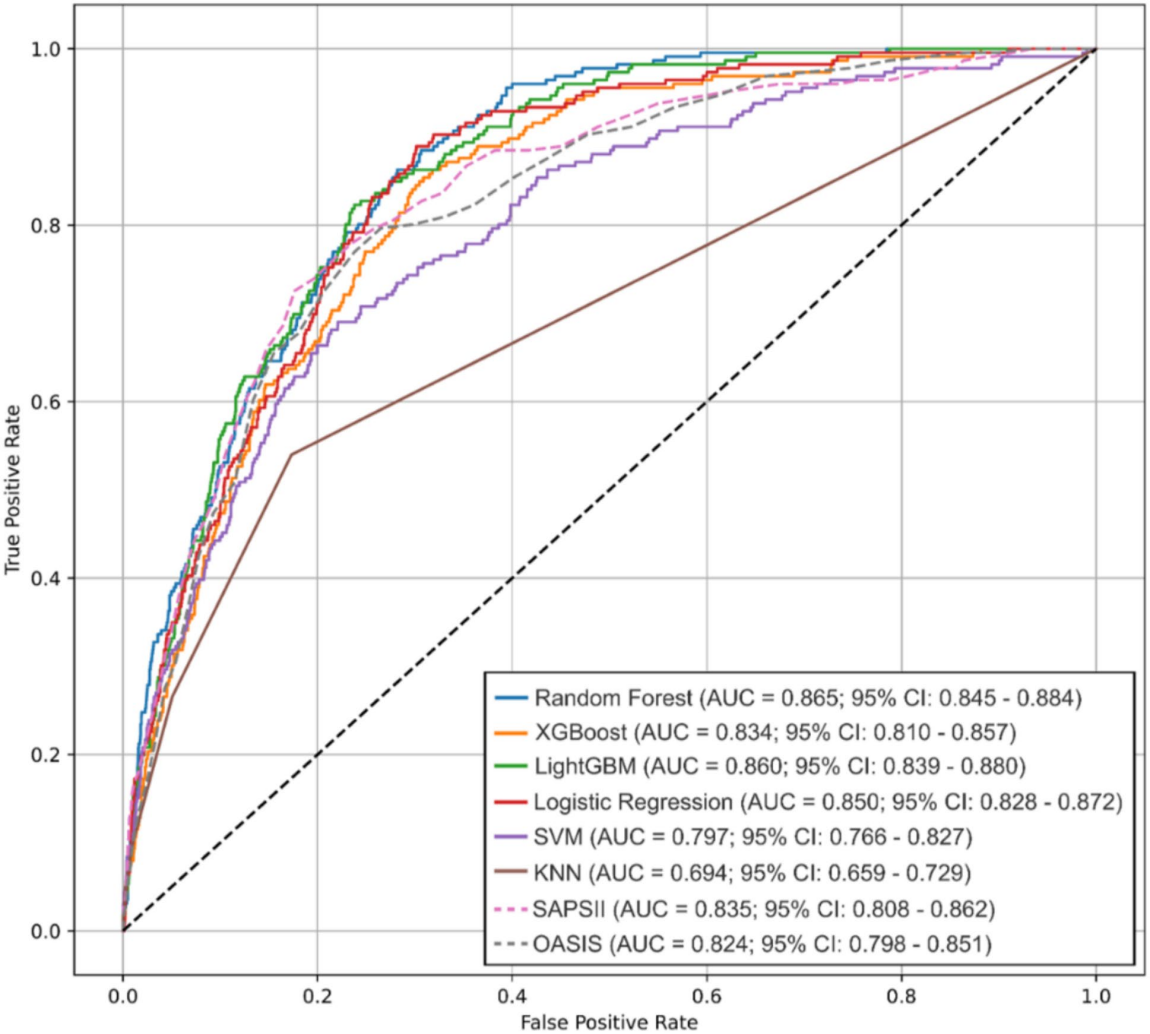
ROC curves for machine learning models

Based on the variables selected by LASSO and Boruta, clinical features including age, mechanical ventilation, vasopressor use, propofol use, renal function status, sepsis, white blood cell count, hemoglobin, platelet count, potassium, and bicarbonate were incorporated into the machine learning analyses (Fig. 4). The Random Forest model showed the best overall performance, with an AUC of 0.865 (95% CI 0.845–0.885), a Brier score of 0.0547, and an accuracy of 0.930 (Table S6). The AUCs for the other models were 0.860 (95% CI 0.839–0.880) for LightGBM, 0.850 (95% CI 0.828–0.872) for Logistic Regression, 0.834 (95% CI 0.814–0.854) for XGBoost, 0.824 (95% CI 0.803–0.844) for OASIS, 0.835 (95% CI 0.808–0.862) for SAPS II, 0.797 (95% CI 0.774–0.820) for SVM, and 0.694 (95% CI 0.659–0.729) for KNN (Fig. 5). SHAP values for each variable are shown in Figure S4. Further comparisons indicated that the predictive performance of the Random Forest model was significantly superior to traditional SAPS II and OASIS scores (DeLong test, *p* < 0.05).

## Discussion

This study utilized the MIMIC database to classify the 24-h temperature trajectories of TBI patients following ICU admission into three distinct subtypes. Class 1 exhibited persistently elevated temperatures, which may reflect an excessive systemic inflammatory response, infection-related hyperthermia, or neurogenic fever secondary to hypothalamic injury. These patients demonstrated intermediate outcomes. Class 2 maintained relatively stable and normal temperature ranges, representing patients with preserved thermoregulatory function and balanced systemic homeostasis, who exhibited the most favorable clinical outcomes. Class 3 was characterized by initial hypothermia followed by rapid temperature elevation, a pattern suggestive of severe central thermoregulatory dysfunction [12]. The subsequent rewarming phase may also be influenced by passive warming measures, such as heating blankets. This subtype was associated with the highest mortality risk, underscoring the clinical significance of early temperature instability as a marker of physiological deterioration.

Traumatic brain injury, characterized by high complexity and heterogeneity, often results in poor clinical outcomes. Previous studies report a mortality rate of approximately 10% among emergency TBI patients, rising to nearly 30% in those admitted to the ICU. Consequently, early identification of high-risk patients has become a central focus in TBI research. For instance, recent evidence indicates that initial whole-head CT perfusion provides substantial predictive value for in-hospital mortality among severe TBI patients [13]. In addition, elevated peak serum sodium [14] and increased glycemic variability (GV) [15] within the first 24 h of admission have been independently associated with in-hospital mortality. Body temperature, a readily and continuously measurable physiological parameter in the ICU, is cost-effective, easy to monitor, and closely linked to secondary brain injury and inflammatory responses. Building on this, the present study analyzes 24-h temperature trajectories following ICU admission to further elucidate the clinical significance of distinct temperature patterns and their relationship with patient outcomes.

The LCMM is a statistical approach capable of simultaneously accounting for inter-individual heterogeneity and intra-individual temporal dynamics, and it has now been widely applied in various clinical studies [7, 16, 17]. Compared with traditional linear mixed models or latent class growth analysis (LCGA), LCMM offers greater flexibility and realism in model specification [10]. It accommodates nonlinear trajectories, asymmetric distributions, and complex time-dependent structures while incorporating random effects to allow for individual variability within the same latent class. This enables a more accurate depiction of physiological changes over time. The key innovation of LCMM lies in its ability to move beyond single timepoint or mean-trend analyses, uncovering latent trajectory subgroups within a population based on longitudinal data. In the present study, LCMM was employed to analyze temperature trajectories during the first 24 h of ICU admission in patients with TBI, successfully identifying latent subtypes with distinct temporal patterns. Such an approach provides a novel statistical tool for early risk stratification in TBI, offering clinicians insights to identify high-risk patients at an early stage of ICU care.

Fever is common in TBI patients within hours to days after injury [18]. Both early hypothermia (< 37 °C) and hyperthermia (> 39 °C) during the first 24 h of ICU admission are associated with increased in-hospital mortality [19]. Moreover, neurogenic fever (core temperature > 37.5 °C) in the absence of infection has been linked to adverse prognosis [20]. Our subgroup analyses further demonstrated that in non-septic patients, the hyperthermic trajectory was associated with poorer outcomes compared with the normothermic trajectory. One possible mechanism involves hypothalamic injury leading to thermoregulatory dysfunction [21]. Age-dependent differences in the impact of fever have been reported [22]. Consistent with this, in this study, the association between hyperthermic trajectories and ICU mortality was observed only in patients aged ≥ 65 years, potentially reflecting age-related susceptibility to cerebral edema [23]. Experimental evidence further supports the deleterious effects of hyperthermia: activation of JNK promotes astrocyte apoptosis after trauma [24], while both mild hypothermia and hyperthermia (≥ 39 °C) impair the transport of anti-inflammatory and antioxidant recombinant HDL into the brain, with hyperthermia additionally inhibiting neuronal uptake of HDL [3]. Early fever may also precipitate paroxysmal sympathetic hyperactivity (PSH) [25] and intracranial hypertension [26].

In addition, hypothermia, acidosis, and coagulopathy—collectively known as the trauma triad of death—are critical predictors of mortality in trauma patients [27]. Correspondingly, laboratory tests in Class 3, which had the lowest initial temperatures, revealed pH < 7.35 and bicarbonate levels < 22, providing further explanation for their unfavorable prognosis.

Therefore, targeted temperature management (TTM), encompassing both therapeutic hypothermia (TH) and normothermia management, is widely recognized as an integral component of high-quality care for patients with TBI. Several studies have demonstrated that TH can reduce mortality [28] in critically ill TBI patients without adversely affecting outcomes in those with post-TBI coagulopathy [29]. Mechanistically, TH may confer neuroprotection by upregulating astrocyte-specific BDNF expression [30]. Nevertheless, its efficacy remains contentious [31], as some reports suggest that TTM does not significantly improve clinical outcomes [32, 33]. Consequently, further prospective clinical trials and preclinical investigations are warranted to elucidate the role of TH in TBI. Furthermore, our results may provide a framework for individualized TTM strategies. Patients in the hyperthermic trajectory (Class 1) may benefit from early and proactive temperature control to mitigate the effects of excessive systemic inflammation or neurogenic fever. Those in the hypothermic trajectory (Class 3) may require careful, gradual rewarming while monitoring for secondary complications, such as acidosis or coagulopathy. Patients in the stable normothermia trajectory (Class 2) may require routine maintenance of normal temperature, avoiding unnecessary interventions. By integrating trajectory information into bedside management, clinicians could tailor temperature interventions to each patient’s physiological pattern, potentially improving outcomes and optimizing resource utilization.

International expert consensus recommends continuous temperature monitoring in TBI patients, with prompt identification and management of fever, particularly in those at risk for secondary brain injury. Maintaining controlled normothermia (36.0–37.5 °C) is suggested as a therapeutic option while emphasizing that temperature management should be individualized based on the risk of secondary injury and the underlying cause of fever [34]. In recent years, machine learning has been increasingly applied to individualized prognostic assessment and risk stratification in TBI, with multiple studies demonstrating its utility in predicting patient outcomes [35–37]. Compared with traditional statistical models, machine learning can handle complex, high-dimensional data and offers enhanced predictive accuracy and interpretability. In this study, we employed trajectory analysis to identify latent subgroups of TBI patients, followed by variable selection using BORUTA and LASSO, and developed six predictive models, among which the random forest model exhibited the best performance. These trajectory-based models provide valuable insights into early temperature dynamics in TBI patients and may inform risk stratification and individualized clinical management.

Several limitations warrant consideration. Although body temperature was assessed, the measurement sites were not uniform across patients. Temperatures in both databases were obtained from multiple anatomical sites (e.g., oral, rectal, bladder, core, and axillary), and nearly half of the records in the eICU database lacked site information. These differences in measurement modality may introduce systematic variability and potentially influence the derived temperature trajectories. In addition, cerebral temperature, which may exceed core body temperature due to the brain’s high metabolic activity [38], was not measured. The absence of normal daily brain temperature rhythms in TBI patients has been linked to a 21-fold increase in ICU mortality [39], underscoring the need for more precise and continuous cerebral temperature monitoring. Finally, cause-specific mortality data (e.g., neurological vs. systemic causes) were not recorded in either the MIMIC-IV or eICU Collaborative Research Databases. As a result, our analysis was limited to all-cause ICU and in-hospital mortality. This restriction precluded further differentiation of deaths primarily attributable to neurological deterioration vs. systemic complications, such as infection or multiorgan failure. Future prospective studies with detailed cause-of-death data and integrated neurophysiological monitoring could help clarify the specific mechanisms linking temperature dysregulation to adverse outcomes in TBI. In addition, while we employed the LCMM framework to identify temperature trajectories, alternative modeling approaches could also be considered. Methods such as growth mixture modeling (GMM) or time-series clustering techniques like dynamic time warping (DTW) may offer complementary insights by capturing different aspects of longitudinal heterogeneity. Although LCMM provides a robust framework that accounts for both inter-individual variability within classes and between-class differences, future studies could benefit from comparing these methods to further validate and generalize the identified trajectory patterns.

## Conclusion

In summary, this study leveraged multiple clinical databases to characterize early temperature trajectories and their associations with clinical outcomes in TBI patients. Three distinct trajectory patterns were identified: initially elevated followed by a gradual decline (Class 1), stable normothermia (Class 2), and initial hypothermia with rapid rewarming (Class 3). These trajectories reflect different patterns of thermoregulatory function and were associated with variations in illness severity and mortality. Our findings indicate that temperature trajectory patterns may serve as informative indicators of disease progression and could help refine risk assessment and guide individualized management in TBI care.

## Supplementary Information

Supplementary Material 1.

## Data Availability

The datasets presented in the current study are available in the MIMIC-IV database (https://physionet.org/content/mimiciv/3.1/) and the eICU database (https://physionet.org/content/eicu-crd/2.0/).
