# Supplementary material for: Influence of early temperature trajectories on clinical outcomes in traumatic brain injury: a multicenter validation study using machine learning
Source: Eur J Med Res. 2025 Dec 2;31:31. doi: 10.1186/s40001-025-03587-z (PMC12777018; doi:10.1186/s40001-025-03587-z)
Supplement: Supplementary file 1 — Supplementary Material 1. [file 40001_2025_3587_MOESM1_ESM.docx]

Table S1. ICD Codes for disease

| Disease | ICD-9 | ICD-10 |
| --- | --- | --- |
| Traumatic brain injury | 800.0–801.9, 850.0–854.1, 950.1–950.3, 995.55, 959.01 | S02.0, S02.1, S02.8, S02.91, S04.02, S04.03, S04.04, S06, S07.1, T74.4 |

Table S2. Baseline characteristics of the original cohort derived from the MIMIC database.

|  | **Class 1 (N=702)** | **Class 2 (N=2481)** | **Class 3 (N=66)** | **p-value** | **SMD (Compare to class1, respectively)** | **Missing data (%)** |
| --- | --- | --- | --- | --- | --- | --- |
| Age | **55.00 [33.25, 73.00]** | **70.00 [53.00, 82.00]** | **56.50 [30.25, 69.00]** | **<0.001** | **[0.567, 0.096]** | **0.00** |
| Gender (Female) | **201 (28.63%)** | **935 (37.69%)** | **19 (28.79%)** | **<0.001** | **[0.193, 0.003]** | **0.00** |
| SOFA score | **4.00 [2.00, 5.00]** | **3.00 [2.00, 4.00]** | **7.00 [4.25, 10.00]** | **<0.001** | **[0.273, 1.002]** | **0.00** |
| Charlson score | **2.00 [0.00, 4.00]** | **4.00 [2.00, 6.00]** | **2.00 [0.00, 4.00]** | **<0.001** | **[0.438, 0.026]** | **0.00** |
| GCS score | **14.00 [10.00, 15.00]** | **14.00 [13.00, 15.00]** | **15.00 [11.25, 15.00]** | **<0.05** | **[0.188, 0.041]** | **0.00** |
| **Interventions (boolean for 1st 24 h)** |  |  |  |  |  |  |
| Mechanical ventilation use (YES) | **508 (72.36%)** | **778 (31.36%)** | **60 (90.91%)** | **<0.001** | **[0.900, 0.493]** | **0.00** |
| RRT use (YES) | **2 (0.28%)** | **23 (0.93%)** | **4 (6.06%)** | **<0.001** | **[0.083, 0.334]** | **0.00** |
| Propofol use (YES) | **438 (62.39%)** | **680 (27.41%)** | **49 (74.24%)** | **<0.001** | **[0.751, 0.257]** | **0.00** |
| Vasopressor use (YES) | **145 (20.66%)** | **253 (10.20%)** | **41 (62.12%)** | **<0.001** | **[0.293, 0.928]** | **0.00** |
| **Comorbidities (boolean)** |  |  |  |  |  |  |
| Renal (YES) | **42 (5.98%)** | **307 (12.37%)** | **6 (9.09%)** | **<0.001** | **[0.223, 0.118]** | **0.00** |
| COPD (YES) | **32 (4.56%)** | **188 (7.58%)** | **3 (4.55%)** | **<0.05** | **[0.127, 0.001]** | **0.00** |
| CAD (YES) | **73 (10.40%)** | **465 (18.74%)** | **5 (7.58%)** | **<0.001** | **[0.238, 0.099]** | **0.00** |
| Stroke (YES) | **71 (10.11%)** | **179 (7.21%)** | **3 (4.55%)** | **<0.05** | **[0.103, 0.215]** | **0.00** |
| Sepsis (YES) | **449 (63.96%)** | **916 (36.92%)** | **49 (74.24%)** | **<0.001** | **[0.562, 0.224]** | **0.00** |
| **Vital signs (1st 24 h)** |  |  |  |  |  |  |
| MAP | 86.00 [75.25, 100.00] | 88.00 [78.00, 99.00] | 81.50 [66.00, 104.75] | 0.15 | [0.020, 0.173] | 0.00 |
| Heart rate | **93.00 [79.00, 107.00]** | **82.00 [71.00, 95.00]** | **87.00 [72.00, 106.00]** | **<0.001** | **[0.473, 0.194]** | **0.00** |
| Temperature | **37.33 [36.89, 37.89]** | **36.78 [36.50, 37.06]** | **34.44 [33.40, 35.46]** | **<0.001** | **[0.889, 2.160]** | **0.00** |
| **Laboratory tests (1st 24 h)** |  |  |  |  |  |  |
| WBC | **12.10 [9.40, 15.60]** | **9.90 [7.43, 13.00]** | **10.55 [6.90, 14.45]** | **<0.001** | **[0.338, 0.249]** | **0.12** |
| Hemoglobin | **11.80 [10.40, 13.20]** | **11.40 [9.90, 12.80]** | **10.15 [8.70, 11.40]** | **<0.001** | **[0.173, 0.576]** | **0.12** |
| Platelet | **205.00 [160.00, 256.00]** | **195.00 [152.00, 250.00]** | **137.00 [97.25, 211.00]** | **<0.001** | **[0.092, 0.537]** | **0.22** |
| Sodium | 139.00 [137.00, 142.00] | 139.00 [136.00, 141.00] | 139.00 [135.25, 141.00] | 0.07 | [0.100, 0.197] | 0.06 |
| Potassium | 4.00 [3.60, 4.30] | 4.00 [3.70, 4.40] | 3.90 [3.42, 4.38] | 0.22 | [0.019, 0.121] | 0.06 |
| Bicarbonate | **23.00 [21.00, 25.00]** | **24.00 [21.00, 26.00]** | **20.00 [17.25, 22.00]** | **<0.001** | **[0.117, 0.724]** | **0.06** |
| Chloride | **104.00 [101.00, 108.00]** | **104.00 [101.00, 107.00]** | **106.50 [103.00, 113.00]** | **<0.001** | **[0.179, 0.379]** | **0.06** |
| BUN | **14.00 [10.00, 18.00]** | **15.00 [11.00, 22.00]** | **15.00 [12.00, 21.00]** | **<0.001** | **[0.200, 0.195]** | **0.22** |
| pH | **7.39 [7.33, 7.44]** | **7.38 [7.33, 7.43]** | **7.27 [7.13, 7.33]** | **<0.001** | **[0.044, 1.103]** | **41.52** |
| PO2 | 162.50 [103.75, 251.00] | 166.00 [98.00, 241.00] | 176.50 [98.00, 364.75] | 0.21 | [0.067, 0.241] | 58.97 |
| PCO2 | **39.00 [34.00, 44.25]** | **39.00 [34.00, 45.00]** | **45.50 [38.75, 55.50]** | **<0.001** | **[0.092, 0.775]** | **60.05** |
| Alkaline phosphatase | **73.00 [57.00, 99.00]** | **77.00 [58.00, 103.00]** | **55.00 [45.00, 80.00]** | **<0.001** | **[0.088, 0.298]** | **59.03** |
| **Outcomes (boolean)** |  |  |  |  |  |  |
| ICU mortality (Death) | **77 (10.97%)** | **125 (5.04%)** | **27 (40.91%)** | **<0.001** | **[0.220, 0.727]** | **0.00** |
| In-hospital mortality (Death) | **116 (16.52%)** | **215 (8.67%)** | **29 (43.94%)** | **<0.001** | **[0.239, 0.625]** | **0.00** |
| Day28 mortality (Death) | **132 (18.80%)** | **333 (13.42%)** | **30 (45.45%)** | **<0.001** | **[0.147, 0.595]** | **0.00** |
| **Length of Stay (days)** |  |  |  |  |  |  |
| ICU length of Stay | **4.49 [2.29, 9.41]** | **2.68 [1.69, 4.93]** | **5.00 [2.05, 16.48]** | **<0.001** | **[0.442, 0.301]** | **0.00** |
| In-hospital length of Stay | **11.13 [5.90, 19.79]** | **6.77 [4.03, 12.76]** | **8.91 [3.77, 26.39]** | **<0.001** | **[0.357, 0.128]** | **0.00** |
| Values are presented as mean (standard deviation) or median [Q1, Q3] for continuous variables and number (percentage) for categorical variables. Variables in bold have p-value < 0.05.  Table S3. Baseline characteristics of the original cohort derived from the eICU database.   \|  \| **class1 (N=712)** \| **class2 (N=2473)** \| **class3 (N=61)** \| **p-value** \| **SMD (Compare to class1, respectively)** \| **Missing data (%)** \| \| --- \| --- \| --- \| --- \| --- \| --- \| --- \| \| Age \| **52.50 [31.00, 70.00]** \| **68.00 [51.00, 81.00]** \| **63.00 [43.00, 78.00]** \| **<0.001** \| **[0.593, 0.343]** \| **0.00** \| \| Gender (Female) \| **205 (28.79%)** \| **1003 (40.56%)** \| **22 (36.07%)** \| **<0.001** \| **[0.249, 0.155]** \| **0.12** \| \| SOFA score \| **7.00 [5.00, 9.00]** \| **5.00 [3.00, 7.00]** \| **9.00 [6.00, 13.00]** \| **<0.001** \| **[0.731, 0.607]** \| **0.00** \| \| Charlson score \| **1.00 [0.00, 3.00]** \| **3.00 [1.00, 5.00]** \| **2.00 [0.00, 4.00]** \| **<0.001** \| **[0.562, 0.333]** \| **0.00** \| \| GCS score \| **1.00 [0.00, 3.00]** \| **3.00 [1.00, 5.00]** \| **2.00 [0.00, 4.00]** \| **<0.001** \| **[0.562, 0.333]** \| **0.00** \| \| **Interventions (boolean for 1st 24 h)** \|  \|  \|  \|  \|  \|  \| \| Mechanical ventilation use (YES) \| **536 (75.28%)** \| **709 (28.67%)** \| **50 (81.97%)** \| **<0.001** \| **[1.055, 0.164]** \| **0.00** \| \| RRT use (YES) \| 3 (0.42%) \| 21 (0.85%) \| 0 (0.00%) \| 0.4 \| [0.054, 0.092] \| 0.00 \| \| Propofol use (YES) \| **328 (46.07%)** \| **381 (15.41%)** \| **18 (29.51%)** \| **<0.001** \| **[0.705, 0.347]** \| **0.00** \| \| Vasopressor use (YES) \| **101 (14.19%)** \| **143 (5.78%)** \| **29 (47.54%)** \| **<0.001** \| **[0.283, 0.774]** \| **0.00** \| \| **Comorbidities (boolean)** \|  \|  \|  \|  \|  \|  \| \| Renal (YES) \| **31 (4.35%)** \| **209 (8.45%)** \| **4 (6.56%)** \| **<0.01** \| **[0.168, 0.097]** \| **0.00** \| \| COPD (YES) \| **31 (4.35%)** \| **211 (8.53%)** \| **4 (6.56%)** \| **<0.001** \| **[0.171, 0.097]** \| **0.00** \| \| CAD (YES) \| **75 (10.53%)** \| **455 (18.40%)** \| **9 (14.75%)** \| **<0.001** \| **[0.225, 0.127]** \| **0.00** \| \| Stroke (YES) \| **73 (10.25%)** \| **367 (14.84%)** \| **9 (14.75%)** \| **<0.01** \| **[0.139, 0.136]** \| **0.00** \| \| Sepsis (YES) \| **177 (24.86%)** \| **219 (8.86%)** \| **14 (22.95%)** \| **<0.001** \| **[0.438, 0.045]** \| **0.00** \| \| **Vital signs (1st 24 h)** \|  \|  \|  \|  \|  \|  \| \| MAP \| **88.00 [76.00, 100.00]** \| **90.00 [79.00, 100.00]** \| **82.00 [65.00, 96.00]** \| **<0.01** \| **[0.077, 0.273]** \| **0.03** \| \| Heart rate \| **94.00 [79.00, 108.00]** \| **81.00 [70.00, 94.00]** \| **85.00 [67.00, 100.00]** \| **<0.001** \| **[0.584, 0.435]** \| **0.00** \| \| Temperature \| **37.39 [36.78, 37.90]** \| **36.72 [36.40, 37.00]** \| **34.22 [33.40, 35.30]** \| **<0.001** \| **[0.605, 2.221]** \| **0.06** \| \| **Laboratory tests (1st 24 h)** \|  \|  \|  \|  \|  \|  \| \| WBC \| **12.50 [9.70, 16.13]** \| **9.80 [7.40, 12.80]** \| **10.22 [8.42, 16.75]** \| **<0.001** \| **[0.450, 0.126]** \| **4.19** \| \| Hemoglobin \| **11.80 [10.20, 13.30]** \| **11.90 [10.40, 13.40]** \| **10.95 [9.00, 13.00]** \| **<0.01** \| **[0.071, 0.385]** \| **4.59** \| \| Platelet \| **191.00 [148.00, 241.00]** \| **195.00 [153.00, 242.00]** \| **149.00 [106.50, 191.50]** \| **<0.001** \| **[0.022, 0.574]** \| **4.22** \| \| Sodium \| **140.00 [137.00, 142.00]** \| **139.00 [137.00, 141.00]** \| **141.00 [137.00, 144.50]** \| **<0.001** \| **[0.142, 0.306]** \| **3.14** \| \| Potassium \| **3.80 [3.60, 4.10]** \| **3.90 [3.60, 4.20]** \| **3.80 [3.45, 4.20]** \| **<0.01** \| **[0.148, 0.006]** \| **3.05** \| \| Bicarbonate \| **23.00 [20.85, 25.45]** \| **24.30 [22.00, 27.00]** \| **21.00 [16.35, 24.00]** \| **<0.001** \| **[0.377, 0.564]** \| **4.68** \| \| Chloride \| **106.00 [103.00, 110.00]** \| **105.00 [102.00, 108.00]** \| **110.00 [104.00, 114.00]** \| **<0.001** \| **[0.230, 0.389]** \| **3.23** \| \| BUN \| **13.00 [9.00, 18.00]** \| **15.00 [11.00, 21.00]** \| **17.00 [14.00, 24.50]** \| **<0.001** \| **[0.244, 0.506]** \| **3.30** \| \| pH \| **7.40 [7.34, 7.44]** \| **7.39 [7.34, 7.44]** \| **7.33 [7.26, 7.40]** \| **<0.001** \| **[0.005, 0.644]** \| **64.97** \| \| PO2 \| 144.50 [90.70, 209.00] \| 137.50 [89.00, 217.00] \| 143.00 [82.75, 242.95] \| 0.85 \| [0.015, 0.131] \| 64.02 \| \| PCO2 \| **37.00 [32.12, 41.00]** \| **38.00 [33.00, 43.00]** \| **38.85 [34.40, 44.25]** \| **<0.01** \| **[0.195, 0.362]** \| **63.89** \| \| Alkaline phosphatase \| **68.00 [55.00, 87.00]** \| **73.00 [58.00, 95.00]** \| **53.00 [45.50, 74.00]** \| **<0.001** \| **[0.115, 0.265]** \| **46.70** \| \| **Outcomes (boolean)** \|  \|  \|  \|  \|  \|  \| \| ICU mortality (Death) \| **91 (12.78%)** \| **111 (4.49%)** \| **24 (39.34%)** \| **<0.001** \| **[0.299, 0.635]** \| **0.00** \| \| In-hospital mortality (Death) \| **129 (18.12%)** \| **196 (7.93%)** \| **35 (57.38%)** \| **<0.001** \| **[0.307, 0.905]** \| **0.96** \| \| Day28 mortality (Death) \|  \|  \|  \|  \|  \|  \| \| **Length of Stay (days)** \| **4.94 [2.47, 10.53]** \| **2.48 [1.63, 4.44]** \| **2.59 [1.55, 7.90]** \| **<0.001** \| **[0.28, 0.17]** \| **0.00** \| \| ICU length of Stay \| **10.27 [5.07, 18.35]** \| **5.81 [3.45, 10.05]** \| **3.65 [2.18, 9.35]** \| **<0.001** \| **[0.347, 0.393]** \| **0.00** \| \| Values are presented as mean (standard deviation) or median [Q1, Q3] for continuous variables and number (percentage) for categorical variables. Variables in bold have p-value < 0.05. \| \| \| \| \| \| \| | | | | | | |

***Table S4 Descriptive statistics for each identified temperature trajectory group.***

| **Number of Classes** | **Log Likelihood** | **AIC** | **BIC** | **SABIC** | **Entropy** | **Class 1 (%)** | **Class 2 (%)** | **Class 3 (%)** | **Class 4 (%)** | **Class 5 (%)** |
| --- | --- | --- | --- | --- | --- | --- | --- | --- | --- | --- |
| **1** | -27,803.42 | 55,614.84 | 55,639.19 | 55,626.48 | 1.00 | 100.00 |  |  |  |  |
| **2** | -25,487.99 | 50,991.98 | 51,040.67 | 51,015.25 | 0.80 | 74.18 | 25.82 |  |  |  |
| **3** | **-23,758.02** | **47,540.04** | **47,613.07** | **47,574.94** | **0.90** | **21.61** | **76.36** | **2.03** |  |  |
| **4** | -23,027.33 | 46,086.67 | 46,184.04 | 46,133.20 | 0.91 | 21.67 | 72.79 | 3.88 | 1.66 |  |
| **5** | -22,488.04 | 45,016.08 | 45,137.80 | 45,074.25 | 0.90 | 4.00 | 0.68 | 71.19 | 18.44 | 5.69 |

***Table S5 Mean of posterior probabilities in each class.***

| **Number of Classes** | **probability 1** | **probability 2** | **probability 3** |
| --- | --- | --- | --- |
| **1** | **0.985** | 0.0000274 | 0.0153 |
| **2** | 0.00000350 | **0.987** | 0.0132 |
| **3** | 0.00660 | 0.139 | **0.854** |

***Table S6 Performance of different predictive models in terms of discrimination and calibration***

| **Model** | **AUC** | **95% CI** | **Brier Score** | **Accuracy** |
| --- | --- | --- | --- | --- |
| XGBoost | 0.834 | 0.814–0.854 | 0.0625 | 0.923 |
| SVM | 0.797 | 0.774–0.820 | 0.0601 | 0.926 |
| KNN | 0.694 | 0.659–0.729 | 0.0611 | 0.932 |
| Random Forest | 0.865 | 0.845–0.885 | 0.0547 | 0.930 |
| OASIS | 0.824 | 0.803–0.844 | 0.0632 | 0.922 |
| Logistic Regression | 0.850 | 0.828–0.872 | 0.0568 | 0.930 |
| LightGBM | 0.860 | 0.839–0.880 | 0.0566 | 0.930 |
| SAPSII | 0.835 | 0.808–0.862 | 0.0733 | 0.904 |


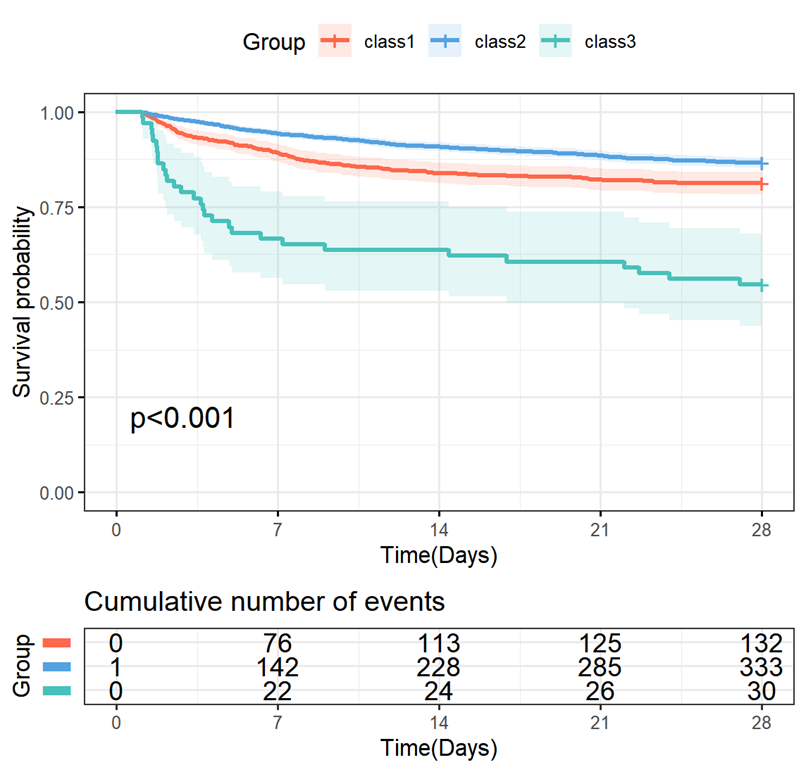


**Figure S1** Kaplan-Meier analysis of 28-day mortality between groups in the MIMIC database.


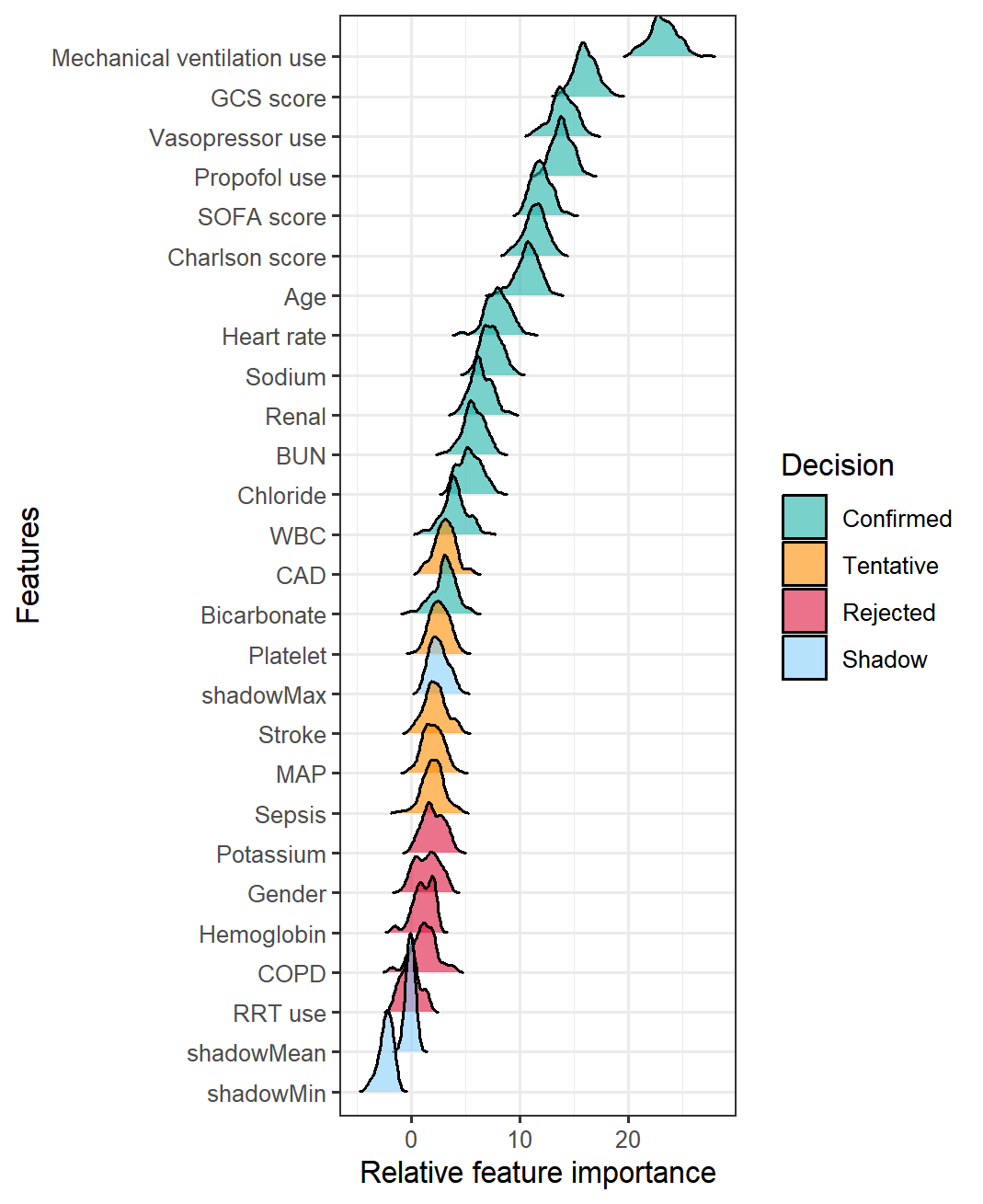


**Figure S2**  Feature selection in eICU database using the Boruta algorithm.

**
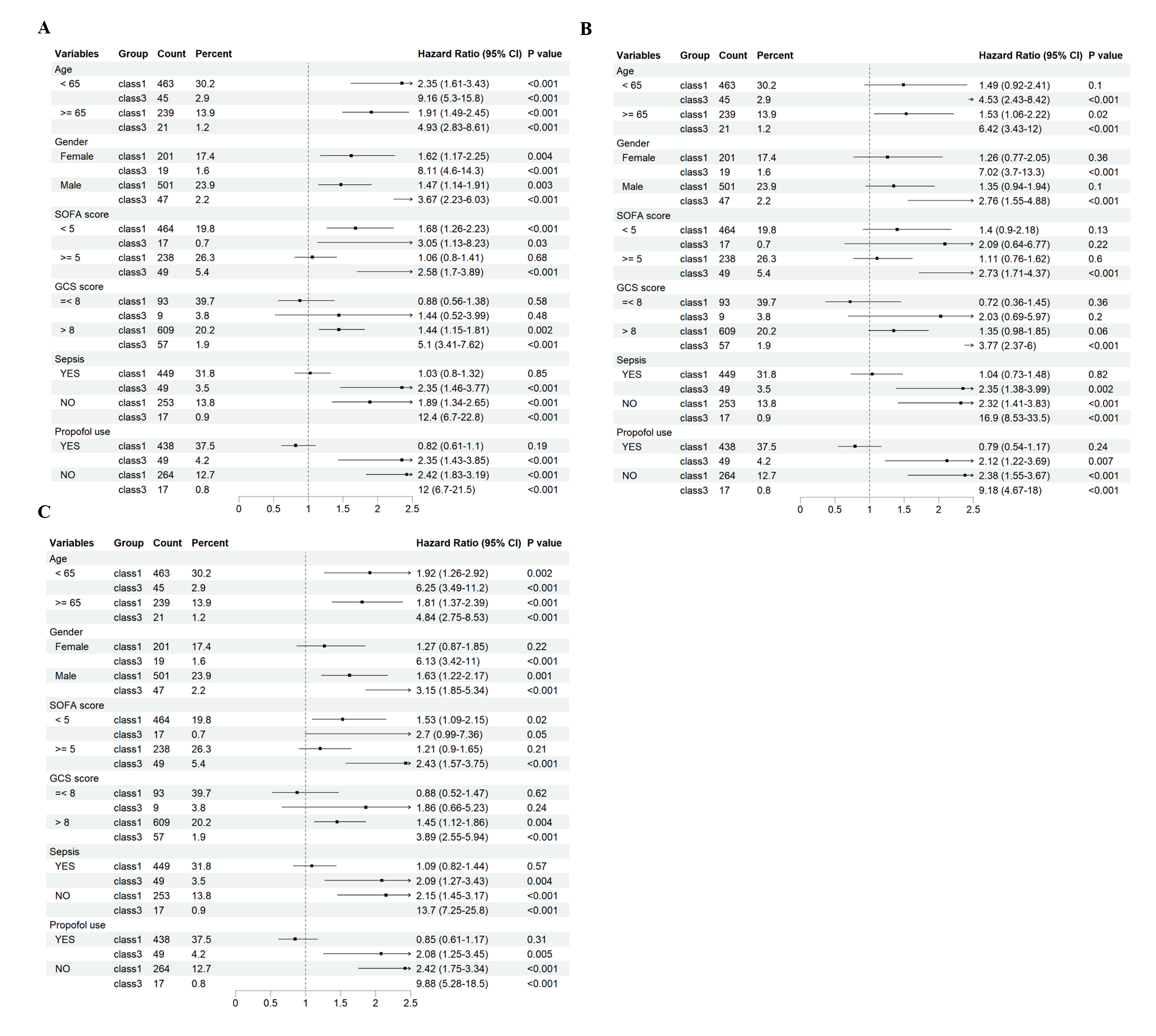
**

**Figure S3** Subgroup analysis of the association between temperature and 28-day mortality (A), ICU mortality (B), and in-hospital mortality (C) in the MIMIC database

**
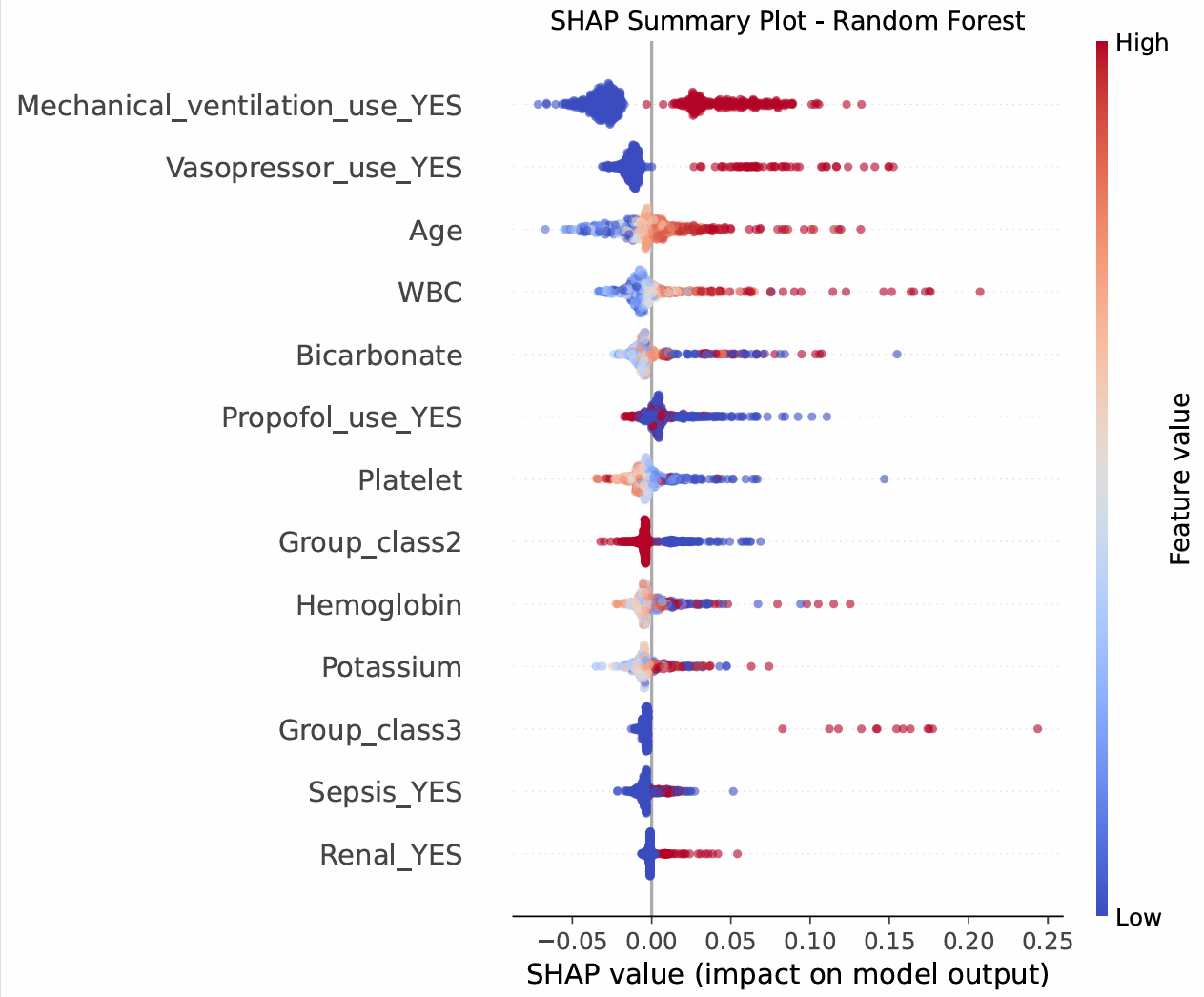
**

**Figure S4** SHAP summary plot showing the impact of each feature on random forest model predictions.
